# Exercise Induces White Adipose Tissue Browning Across the Weight Spectrum in Humans

**DOI:** 10.3389/fphys.2018.01781

**Published:** 2018-12-11

**Authors:** Berenice Otero-Díaz, Marcela Rodríguez-Flores, Verónica Sánchez-Muñoz, Fernando Monraz-Preciado, Samuel Ordoñez-Ortega, Vicente Becerril-Elias, Guillermina Baay-Guzmán, Rodolfo Obando-Monge, Eduardo García-García, Berenice Palacios-González, María Teresa Villarreal-Molina, Mauricio Sierra-Salazar, Barbara Antuna-Puente

**Affiliations:** ^1^Laboratorio de Genómica de Enfermedades Cardiovasculares, Instituto Nacional de Medicina Genómica, Mexico City, Mexico; ^2^Departamento de Endocrinología, Clínica de Obesidad y Trastornos de la Conducta Alimentaria, Instituto Nacional de Ciencias Médicas y Nutrición Salvador Zubiran, Mexico City, Mexico; ^3^Centro de Nutrición y Obesidad, The American British Cowdray (ABC) Medical Center, Mexico City, Mexico; ^4^Departamento de Cirugía, Servicio de Cirugía Endocrina y Laparoscopia Avanzada, Instituto Nacional de Ciencias Médicas y Nutrición Salvador Zubiran, Mexico City, Mexico; ^5^Unidad de Investigación en Enfermedades Oncológicas, Hospital Infantil de México Federico Gómez, Mexico City, Mexico; ^6^Unidad de Vinculación Científica, Instituto Nacional de Medicina Genómica, Mexico City, Mexico

**Keywords:** physical activity, adipose tissue, browning, insulin sensitivity, adipokines, myokines

## Abstract

While the effect of exercise on white adipose tissue browning and metabolic improvement in rodents is clear, there are few studies in humans with inconclusive results. Thus, the aim of the study was to assess whether an exercise intervention promotes subcutaneous adipose tissue browning in humans, and whether this response is associated with metabolic improvement in three groups of individuals defined by body mass index (BMI) (kg/m^2^). Sedentary adult subjects with different BMI were enrolled in a 12-week bicycle-training program (3 times per week, intensity 70–80% HRmax). Brown and beige gene expression in subcutaneous adipose tissue (scWAT) biopsies, and serum glucose, insulin, lipid, adipokine, and myokine levels were compared before and after the exercise intervention. Thirty-three non-diabetic subjects (mean age 30.4 ± 4.6 years; 57.57% female; 13 normal weight, 10 overweight and 10 with obesity) completed the exercise intervention. Without any significant change in body composition, exercise improved several metabolic parameters, most notably insulin resistance and particularly in the overweight group. Circulating adiponectin, apelin, and irisin exercise-induced changes predicted 60% of the insulin sensitivity improvement. After exercise *UCP1, TBX1, CPT1B* scWAT expression significantly increased, along with P2RX5 significant positive staining. These changes are compatible with scWAT browning, however, they were not associated with glucose metabolism improvement. In conclusion, 12-weeks of exercise training produced brown/beige gene expression changes in abdominal scWAT of non-diabetic individuals with different BMI, which did not contribute to the metabolic improvement. However, this result should not be interpreted as a lack of effect of browning on metabolic parameters. These findings suggest that a bigger effect is needed and should not preclude the development of more effective strategies of browning. Furthermore, exercise-induced changes in adiponectin, apelin, and irisin predicted insulin sensitivity improvement, supporting the important role of adipokines and myokines in metabolism homeostasis.

## Introduction

Exercise has many health benefits, even in the absence of body weight reduction (Thyfault and Wright, 2016). Among these, the switch from white adipocytes to brown-like adipocytes (beige or brite adipocytes), or browning, has gained considerable attention. Because the amount and metabolic activity of brown adipose tissue (BAT) vary greatly in humans (Cypess et al., 2009; Saito et al., 2009), strategies aimed at activating existent BAT may be less effective in individuals with low BAT amounts (Elattar and Satyanarayana, 2015). As white adipose tissue (WAT) is more abundant, strategies aimed at browning adipose tissue could induce considerably more effective energy expenditure.

The exact mechanisms by which WAT undergoes browning during exercise are not well understood, however, there is evidence that increased sympathetic innervation of subcutaneous WAT (Bartness and Ryu, 2015), and exercise-induced release of hormones and cytokines such as catecholamines, fibroblast growth factor 21 (FGF21), IL-6, irisin, meteorin-like 1 and myostatin may play a role in this process (Pedersen and Febbraio, 2012). In rodents, exercise training was shown to induce WAT adaptation by increasing thermogenic and oxidative capacities: swimming increased mitochondrial biogenesis, mitochondrial DNA content and glucose uptake in WAT (Trevellin et al., 2014), while running wheel increased *UCP1, PRM16, CIDEA, DIO2, PGC1α* gene expression and improved glucose metabolism (Cao et al., 2011; Boström et al., 2012; Stanford et al., 2015a). Although studies in the murine model have consistently shown that exercise induces WAT browning and glucose homeostasis improvement and that browning has a protective effect against diet-induced obesity, few studies have been performed in humans. In most of these studies little or no changes in WAT browning or oxidative markers were observed and the effect of exercise training on adipose tissue browning in humans remains unclear. These studies vary in gender, age, and health status of participants, also in exercise type, frequency, intensity and duration of the intervention, and methods used to assess WAT browning (Camera et al., 2010; Norheim et al., 2014; Nakhuda et al., 2016; Pino et al., 2016; Stinkens et al., 2018; Tsiloulis et al., 2018). It is known that browning capacity decreases with age (Berry et al., 2017), and could be dependent of the intensity and the duration of the exercise. The nature of the stimulus is important because it induces only a transient *UCP1* expression in beige adipocytes, ceasing upon stimulus withdrawal (Petrovic et al., 2010; Wu et al., 2012; Altshuler-Keylin et al., 2016). We thus hypothesized that a controlled and prolonged endurance exercise training program will induce browning of subcutaneous white adipose tissue (scWAT) in non-diabetic young adults, and explored whether this response differed according to BMI.

## Materials and Methods

### Study Design and Participants

In this non-randomized, controlled trial, non-diabetic subjects of both sexes aged between 20 and 40 years, BMI between 20 and 40 kg/m^2^ and sedentary (no purposeful exercise) for at least the three previous months, were not taking any medication, were recruited using advertisements and referrals from healthcare providers. Potential participants were excluded if they had significant illness (hepatic or renal failure, diabetes, cancer or any condition preventing physical activity), and more than 10% body weight change in the preceding six months. Women were ineligible if they were pregnant or breastfeeding.

The study population was categorized in three groups according to BMI: normal weight (Nw) with BMI between 18 and 24.9 kg/m^2^; overweight (Ow) with BMI between 25 and 29.9 kg/m^2^; and obesity group (Ob) with a BMI between 30 and 40 kg/m^2^.

### Ethical Approval

The protocol was approved by the Instituto Nacional de Ciencias Médicas y Nutrición “Salvador Zubirán” IRB/IEC (#1040), in accordance with the Declaration of Helsinki (13/LO/0078). All participants provided written informed consent.

### Intervention

Exercise intervention consisted of 60 min of supervised aerobic exercise sessions, three days per week, for 12 weeks in accordance with the American Heart Association, the American College of Sports Medicine and the World Health Organization global recommendations for physical activity (Garber et al., 2011). Each exercise session was divided in three stages: 10 min warm up, 40 min of aerobic activity in a recumbent stationary bicycle (Life Cycle OSR, Life Fitness), and 10 min stretching and cool-down. The first three weeks consisted of physical conditioning: 15 min of continuous exercise at an intensity of 60–65% maximum heart rate (HRMax) on week 1, three 25 min sessions at 65–70% HRMax on week 2, and three 30 min sessions at 70–75% HRMax on week 3. From week 4 to 12, participants performed 40 min of exercise per session at an intensity of 80% HRMax (mean HR 153 bpm, range 142–162 bpm), three days a week until the conclusion of the program. Heart rate was monitored with a Lifepulse^TM^ with DSP (digital signal processing) device located on the bicycle handlebars, and HRMax was calculated as (220-age). Participants were asked to keep their usual diet and normal daily activity level during the intervention.

### Anthropometric and Biochemical Measurements

After a detailed medical history, weight, height, waist and hip circumference were measured, and BMI and waist to hip ratio were calculated. Weight and height were measured on a standard balance-beam scale with stadiometer (Seca 213, Hamburg, Germany). Body composition was assessed by bioelectrical impedance analysis (BIA) (Tanita BC-418 Segmental Body Composition Analyzer, IL, United States), with a reported CV < 2% (Pietrobelli et al., 2004; Lee et al., 2014).

After eight hours of fasting, venous blood samples were obtained at baseline and 12 weeks after the exercise intervention. In addition, a single blood sample was drawn immediately after having reached 80% HRMax between weeks 4 and 12 for acute response assessment. Serum and plasma were obtained and stored at –80°C until analysis. Glucose was assessed by the glucose oxidase method. Triglycerides, cholesterol, creatinine, aspartate transaminase (AST) and alanine transaminase (ALT) were measured using an enzymatic colorimetric assay (UniCel DxC 600 Synchron Clinical System, Beckman Coulter, Brea, CA, United States). Plasma insulin was determined by radioimmunoassay based on chemiluminescence (Access 2 Immunoassay System, Beckman Coulter, CA, United States). Insulin sensibility was calculated using Homeostasis model of assessment (HOMA-IR).

Plasma irisin was measured by colorimetric enzyme-linked immunosorbent assay (ELISA) (EK-067-29, Phoenix Pharmaceuticals, Burlingame, CA, United States). Leptin, adiponectin, Interleukin-6 (IL-6), FGF21, and apelin were assessed by ELISA kits following manufacture instructions (R&D Systems, Minneapolis, MN, United States; Abnova Products, Taiwan for apelin).

### Adipose Tissue Gene Expression

Subcutaneous adipose tissue biopsies were collected before and after 12 weeks of exercise. All biopsies were obtained by incision under local anesthesia (2% lidocaine/epinephine), 4 cm below the umbilicus. Tissue samples were rinsed with isotonic saline solution and snap frozen in liquid nitrogen.

RNA was isolated from all samples using the RNeasy Lipid Tissue Mini Kit (Qiagen, Germany). cDNA was synthesized using the SuperScript Kit (Invitrogen, Foster City, CA, United States), by both random hexamers and oligo dT. Gene expression levels of white, brown/beige and beige adipose tissue markers were quantified using Taqman probes in a QuantStudio^®^ 7 Flex Real-Time PCR System (Applied Biosystems^®^). White adipose tissue probes included integrin subunit beta 1 (*ITGB1*, Hs00559595_m1), leptin (*LEP*, Hs00174877_m1), homeobox protein Hox-C8 (*HOXC8*, Hs00224073_m1) and homeobox protein Hox-C9 (*HOXC9*, Hs00396786_m1). Brown/beige adipose tissue probes included PR domain zinc finger protein 16 (*PRDM16*, Hs00922674_m1), cell death activator CIDE-A (*CIDEA*, Hs00154455_m1), cytochrome c oxidase subunit 7A1 (*COX7A1*, Hs03045102_g1), type II iodothyronine deiodinase (*DIO2*, Hs00988260_m1), carnitine palmitoyltransferase 1A (*CPT1A*, Hs00912671_m1) and carnitine palmitoyltransferase 1B (*CPT1B*, Hs03046298_s1). Beige adipose tissue probes included T-box transcription factor 1 (*TBX1*, Hs00271949_m1) and transmembrane protein 26 (*TMEM26*, Hs00415619_m1). Irisin gene expression was also assessed with a Taqman probe (fibronectin type III domain-containing protein 5 or *FNDC5*, Hs00401006_m1). Uncoupling protein 1 (*UCP1*) and integrin subunit alpha 4 (*ITGA4*) gene expression were assessed using the LightCycler→ 480 SYBR Green I Master mix (Roche) assay in a LightCycler→ 480 Instrument II (Roche^®^). Fw: 5′-CTCCAGGTCCAAGGTGAATG-3′ and Rv: 5′-TAGAGGCCGATCCTGAGAGA-3′ primers were used for *UCP1*, while Fw: 5′-GCAAGGAAGTTCCAGGTTACA-3′, and Rv: 5′-CACGTCAGAAGTTCCATTAGAAGA-3′ primers were used for *ITGA4* (Sigma-Aldrich, St. Louis, MO, United States). Quantitative measures were obtained using the ΔΔCt method, and were normalized to TATA-binding protein (*TBP*) mRNA expression.

### Adipose Tissue Morphology

The avidin biotin-peroxidase complex method was used for immunostaining of formalin-fixed, paraffin-embedded sections. Sections were deparaffinized and rehydrated. The antigen was retrieved using boiling sodium citrate. Sections were incubated overnight at room temperature with rabbit polyclonal primary antibody anti-UCP1, anti-TBXI, anti-TMEM26 and anti-Cytochrome C, and for different adipocyte superficial markers including amino acid transporter asc-1 (ASC-1), purinergic receptor P2X (P2RX5), proton-coupled and amino acid transporter 2 (PAT2); eosinophil markers CD137, CD38 and Anti-Sicglec8 (Abcam, United Kingdom). The Expose Mouse and Rabbit Specific HRP/DAB Detection IHC Kit was used for detection following manufacture instructions (Abcam, United Kingdom). Staining was achieved adding diaminobenzidine substrate (DAB). Immunohistochemical stains were digitally analyzed by quantitative computerized image software using the Aperio CS scanner (San Diego, CA, United States).

### Statistical Analysis

Pair-sample *t*-test or non-parametric Wilcoxon’s test were used to compare all baseline and after exercise parameters, as deemed appropriate. One-way ANOVA or Kruskal–Wallis were used to compare differences among groups, followed by Bonferroni post hoc testing. ANCOVA and repeated-measures ANCOVA were used to adjust for potential confounders. Pearson or Spearman’s test were used to test for correlations. Multiple linear regression analysis («enter» method) follow by a stepwise analysis was used to identify variables independently associated with insulin resistance. Variables not showing normal distribution were log-transformed for the analyses. All statistical tests were performed using SPSS version 20.0 software (IBM Corp., New York, NY, United States) and GraphPad PRISM 6.0. A *p*-value below 0.05 was regarded as significant.

## Results

### Subjects

Thirty-three subjects were enrolled and completed the 12 weeks intervention (57.5% female, mean age 30.4 ± 4.6 years), 13 Nw, 10 Ow and 10 Ob.

Subcutaneous adipose tissue gene expression was studied in 28 paired biopsies, since five patients did not agree to the second biopsy. Immunohistochemistry was performed only in 23 paired biopsies, due to insufficient tissue sample.

**Table 1:** Anthropometric and biochemical characteristics at baseline and after 12 weeks of exercise intervention.

	Before (*n* = 33)	After (*n* = 33)	*p*
**% Female**	19 (57.57%)		
**IMC (kg/m^2^)**	27.26±5.16	27.01±4.90	0.077
**Weight (kg)**	74.03±17.61	73.31±16.93	0.055
**Waist (cm)**	90.66±14.07	89.31±12.88	<0.01
**Hip (cm)**	101.30±12.18	100.48±11.76	<0.05
**Total Fat (%)**	30.83±5.86	30.51±5.80	NS
**FFM (%)**	69.16±5.86	69.48±5.80	NS
**Glucose (mg/dL)**	92.24±8.89	86.81±9.12	<0.01
**Triglycerides (mg/dL)**	165.72±120.67	129.30±73.93	<0.001
**Cholesterol (mg/dL)**	193.72±43.09	183.27±41.31	<0.05
**HDL-C (mg/dL)**	45.12±10.79	43.67±11.21	NS
**LDL (mg/dL)**	115.45±29.72	112.77±32.15	NS
**Insulin (μU/L)**	10.26±7.71	9.35±7.53	<0.05
**HOMA-IR**	2.42±2.11	2.08±1.79	<0.01
**Irisin (ng/mL)**	9.33±1.56	9.75±1.53	NS
**Acute Irisin (ng/mL)**	9.55±1.39	11.04±1.80	< 0.001
**Leptin (ng/mL)**	21.49±11.48	19.09±10.15	0.054
**Adiponectin (μg/mL)**	7.30±3.81	6.05±2.93	<0.001
**IL-6 (pg/mL)**	4.85±3.33	4.67±3.14	NS
**Acute IL-6 (pg/mL) (*n* = 23)**	4.97±4.00	6.00±3.32	<0.001
**Apelin (ng/mL) (*n* = 19)**	264.66±119.04	169.73±124.76	<0.05
**FGF-21 (pg/mL)**	506.75±309.58	510.45±254.82	NS
**FGF-21 acute (pg/mL) (*n* = 23)**	424.07±201.61	474.32±269.57	NS

*Data are presented as mean ± standard deviation. NS, not significant. Waist adjusted by BMI*.

### Anthropometric and Biochemical Changes

Table 1 shows anthropometric and biochemical parameters for all subjects before and after the exercise intervention. Overall, weight and BMI decreased slightly with only marginal significance (*p* = 0.055 and *p* = 0.077, respectively), while waist and hip circumferences decreased significantly after exercise (*p* < 0.01 and *p* < 0.05, respectively). No significant changes in body composition were observed. In addition, most biochemical parameters improved, as glucose, insulin, triglyceride and total cholesterol levels decreased and insulin sensitivity increased significantly (Table 1).

**Table 2:** Anthropometric and biochemical characteristics at baseline and after 12 weeks of exercise intervention by BMI groups.

	**Normal weight (*n* = 13)**			**Overweight (*n* = 10)**			**Obesity (*n* = 10)**		
	**Baseline**	**After**	***p***	**Baseline**	**After**	***p***	**Baseline**	**After**	***p***
			
**BMI (kg/m2) l,l l**	22.34±1.45	22.27±1.55	NS	27.25±1.47	27.15±1.39	NS	33.67±3.01	33.03±2.79	<0.05
**Waist (cm) l,l l**	78.05±6.45	77.96±6.29	NS	91.19±8.20	89.34±7.02	NS	106.55±8.45	104.04±7.85	<0.01
**Hip (cm) l,l l**	91.00±8.84	90.43±8.80	NS	102.35±4.95	102.11±4.63	NS	113.65±8.68	111.93±8.54	NS
**Glucose (mg/dL)l,l l**	87.92±8.07	81.23±9.68	<0.05	98.20±9.10	88.30±7.33	<0.01	91.90±6.72	92.60±5.69	NS
**Triglycerides (mg/dL) l l**	118.00±55.21	86.30±28.85	<0.01	210.80±180.60	162.20±100.39	0.055	182.70±96.07	152.30±61.53	NS
**Cholesterol (mg/dL) l, l l**	180.76±36.33	166.69±34.85	<0.05	222.00±45.36	211.50±49.71	NS	182.30±38.85	176.60±25.39	NS
**HDL-C (mg/dL)l, l l**	48.15±7.62	46.77±9.93	NS	48.80±14.79	47.50±8.48	NS	37.50±5.01	35.80±12.03	NS
**LDL (mg/dL)**	108.99±26.39	102.66±25.56	0.093	131.04±15.70	131.56±39.49	NS	108.26±39.73	107.14±25.87	NS
**Insulin (μU/L)l, l l**	6.40±2.47	4.69±2.32	<0.001	12.40±11.46	8.60±8.47	<0.05	13.15±6.00	16.16±6.26	NS
**HOMA-IR l, l l**	1.41±0.66	0.95±0.53	<0.001	3.15±3.28	1.91±1.92	<0.01	3.00±1.46	3.73±1.55	NS
**Irisin (ng/mL)**	9.38±1.60	9.71±1.76	NS	9.60±1.25	10.15±1.24	<0.05	9.00±1.88	9.39±1.52	NS
**Acute Irisin (ng/mL)**	9.92±1.52	11.50±2.03	<0.05	9.43±1.35	10.70±1.57	<0.05	9.26±1.31	10.87±1.79	<0.01
**Leptin (ng/mL) l, l l**	14.80±5.66	14.47±5.29	NS	23.00±10.17	17.87±8.02	0.074	28.67±14.08	26.30±13.21	NS
**Adiponectin (μg/mL) l, l l**	9.36±3.95	7.26±2.93	<0.05	7.07±3.31	6.31±2.76	NS	4.86±2.64	4.21±2.37	<0.05
**IL-6 (pg/mL) l, l l**	4.08±1.09	3.89±1.00	NS	4.01±0.56	3.57±0.67	<0.05	6.69±5.68	6.80±5.10	NS
**Acute IL-6 (pg/mL)**	3.98±1.24	5.49±2.63	<0.05	3.91±0.65	4.74±1.16	0.061	7.87±7.33	8.33±5.02	NS
**Apelin (n/mL) (*n* = 19) (Nw = 7 Ow = 6, Ob = 6)**	280.84±129.86	150.68±91.51	<0.05	301.17±113.53	174.69±155.58	NS	209.29±110.66	187.00±144.53	NS
**FGF-21 (pg/mL)**	445.48±238.35	417.48±147.60	NS	459.40±247.01	479.10±215.52	NS	633.76±422.09	662.67±340.67	NS
**Acute FGF-21 (pg/mL) (*n* = 23) (Nw = 10 Ow = 7, Ob = 6)**	371.22±174.85	380.89±183.92	NS	511.79±270.41	558.53±305.52	NS	409.82±139.54	531.78±339.86	NS

*Data are presented as mean standard deviation. Difference between BMI groups l before, l l after *p* < 0.05. NS, not significant. Waist adjusted by BMI*.

Table 2 shows anthropometric and biochemical characteristics stratified by BMI groups. BMI and waist circumference decreased significantly only in the Ob group (*p* < 0.05 and *p* < 0.01, respectively). Although the mean values of all parameters were within the normal range in the Nw group at baseline, fasting glucose, insulin, triglycerides and cholesterol levels decreased and insulin sensitivity improved in a significant manner after the exercise intervention. In contrast, in Ow subjects mean HOMA-IR, triglyceride, total cholesterol and LDL-C values were elevated at baseline, and all parameters significantly improved after intervention except LDL-C levels (Table 2). We found no significant improvement of any biochemical parameter after exercise intervention in the Ob group.

### Circulating Myokine, Adipokine and Cytokine Levels in Response to Exercise

Circulating leptin and adiponectin levels decreased after the 12-week exercise intervention (*p* = 0.054 and *p* < 0.001, respectively) (Table 1). When stratifying by BMI, leptin levels showed no significant change in normal weight and individuals with obesity. Leptin levels decreased in the Ow group, with only marginal significance (*p* = 0.074). While mean adiponectin levels decreased in all groups, the difference was only significant in the Nw and Ob groups (*p* < 0.001 and *p* < 0.05, respectively) (Table 2).

As expected, irisin did not change after 12 weeks of exercise in the entire sample (Table 1), but when stratifying by BMI groups, irisin levels increased significantly only in the Ow group (*p* < 0.05). Mean circulating irisin levels measured immediately after a single exercise session increased significantly in the entire sample (*p* < 0.001), and in all BMI groups. Chronic and acute irisin responses did not differ significantly among groups (Table 2), and circulating irisin levels did not correlate with BMI. No sex differences in baseline or post-exercise intervention irisin levels were observed even after adjusting for FFM. As regard to the acute response, irisin levels increased in both sexes, however, the magnitude of the response was greater and the difference was significant only in men (from 9.75 ± 1.39 to 11.85 ± 2.03 ng/mL; *p* < 0.001).

Mean IL-6 levels were similar in Nw and Ow subjects but significantly higher in Ob group both at baseline and after the 12-week exercise intervention. IL-6 levels decreased significantly only in the Ow group. In contrast, after a single exercise session, IL-6 significantly increased in all subjects (*p* < 0.01) and in all BMI groups, but reached statistical significance only in the Nw group (*p* < 0.05) (Table 2). No sex differences in IL-6 levels were observed at baseline or after 12 weeks of exercise, however, the acute IL-6 response was significantly increased from 4.21 ± 0.98 to 5.54 ± 1.72 pg/mL only in men (*p* < 0.001).

FGF21 serum levels showed no significant changes immediately after a single exercise session or after the 12-week intervention. Moreover, circulating apelin levels decreased after the 12 -week intervention in the whole sample (*p* < 0.05), and in all BMI groups, only reaching statistical significance in the Nw group (Tables 1, 2).

In a stepwise regression analysis, the best predictive model for exercise-induced insulin sensitivity improvement included changes in serum adiponectin [B-coef:0.54 (95% CI:0.030, 0.12)], irisin [B-coef:0.43 (95% CI:0.016, 0.11)] and apelin levels [B-coef:0.37 (95% CI:0.034, 0.50)] after 12 weeks of exercise. These three parameters explained 60% of the insulin sensitivity response to exercise (*p* = 0.001).

**FIGURE 1:**
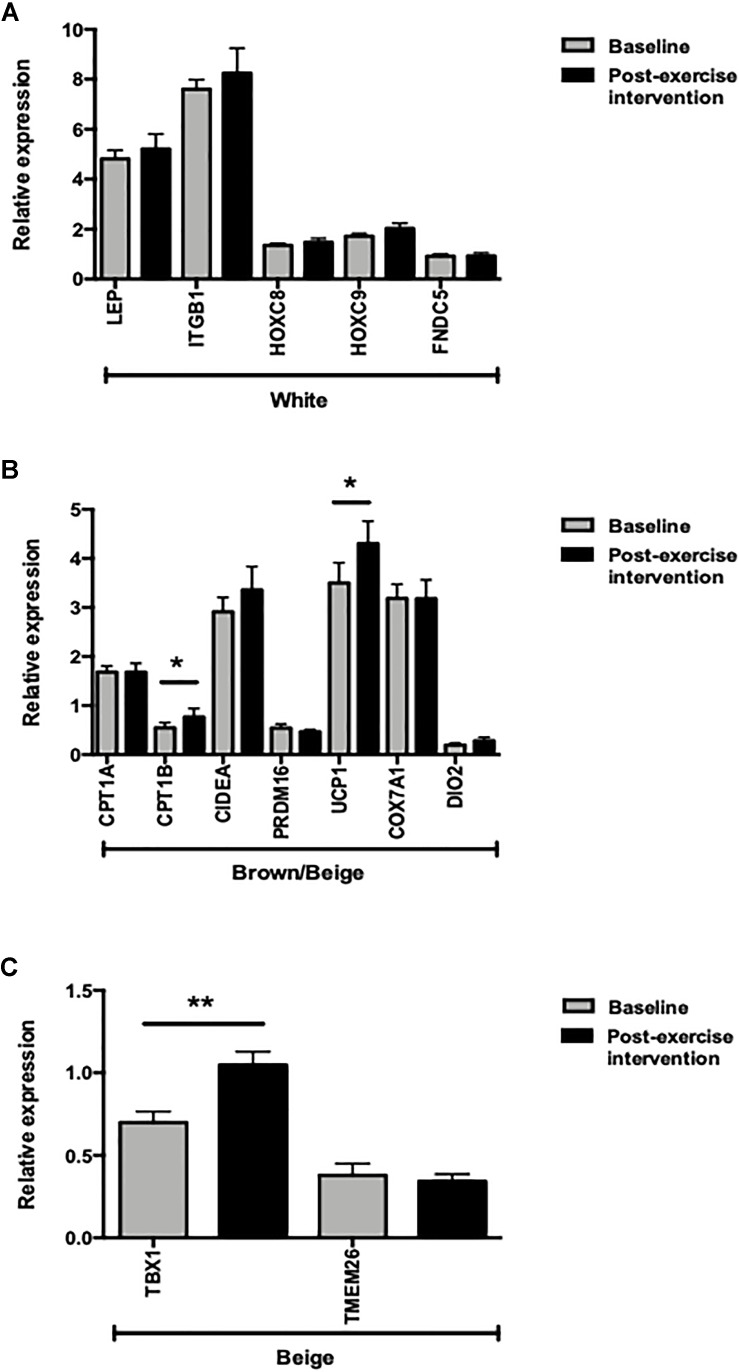
mRNA expression levels of adipose tissue markers before and after 12 weeks of exercise (*n* = 28). **(A)** White adipose tissue markers. **(B)** Brown/beige adipose tissue markers. **(C)** Beige adipose tissue markers. ^∗^*p* < 0.05 and ^∗∗^*p* < 0.01.

### SAT Gene Expression After 12 Weeks of Exercise

In the whole sample, *FNDC5* gene expression levels did not change significantly after the exercise intervention. Similarly, no changes in WAT gene marker expression levels (*LEP, ITGB1, HOXC8* and *HOXC9*) were observed after 12 weeks of exercise (Figure 1A). Among the brown/beige gene markers, only *UCP1* and *CPT1B* expression increased significantly (*p* < 0.05), while the beige adipose tissue marker *TBX1* expression increased significantly after the 12-week exercise intervention (*p* < 0.001) (Figures 1B,C).

**FIGURE 2:**
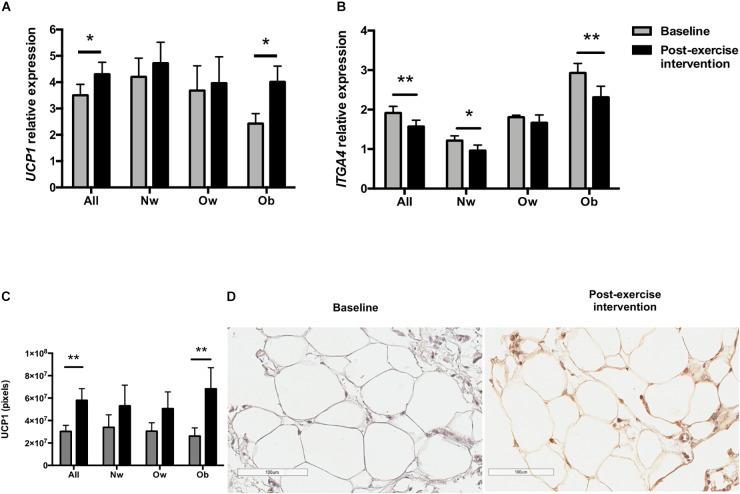
*UCP1* and *ITGA4* expression in scWAT biopsies before (baseline) and after the 12-week exercise intervention. **(A)** *UCP1* mRNA expression according to BMI groups (Nw *n* = 12; Ow *n* = 7; Ob *n* = 9). **(B)** *ITGA4* expression according to BMI groups. **(C)** UCP1 immunostaining intensity according to BMI groups (*n* = 23). **(D)** UCP1 immunohistochemical staining of a representative scWAT image. The magnification for the picture was 100 μm. ^∗^*p* < 0.05 and ^∗∗^*p* < 0.01.

*UCP1* increased in both sexes, only reaching statistical significance in women (*p* < 0.05). Although *UCP1* gene expression increased in all BMI groups, the difference was significant only in the Ob group (*p* < 0.05). Baseline *UCP1* expression levels were the lowest in this group, being significantly different from Nw individuals (*p* < 0.05) (Figure 2A). Interestingly, after the exercise intervention, UCP1 mRNA increased in individuals with obesity (∼1.65 fold), reaching levels comparable to those of Nw group. To assess the hypothesis that *UCP1* gene expression is inhibited by inflammation in obesity (Chung et al., 2017), we measured *ITGA4* gene expression before and after the intervention. *ITGA4* mRNA expression decreased in all the subjects (*p* < 0.001), and in all groups, only reaching significance in Nw (*p* < 0.05) and Ob subjects (*p* < 0.01). *ITGA4* mRNA expression differed among BMI groups both at baseline and after the exercise intervention (*p* < 0.001 and *p* < 0.001, respectively), being higher in the Ob group (Figure 2B).

**FIGURE 3:**
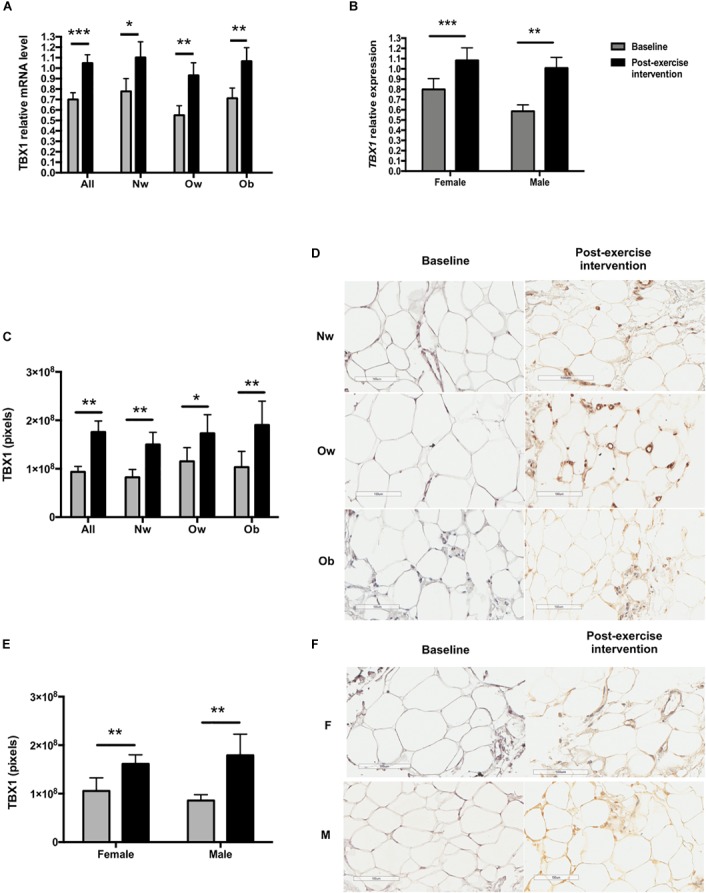
TBX1 expression in scWAT biopsies before and after the exercise intervention according to BMI group and sex. **(A)** *TBX1* mRNA expression according to BMI group (Nw *n* = 12; Ow *n* = 7; Ob *n* = 9). **(B)** *TBX1* mRNA expression according to sex (female *n* = 15; male *n* = 13). **(C)** TXB1 immunostaining intensity according to BMI group (*n* = 23, Nw = 9 Ow = 6 Ob = 8). **(D)** TBX1 immunohistochemical staining of a representative scWAT image of a normal weight, overweight and an obese individual before and after the exercise intervention. **(E)** TBX1 immunostaining total intensity according to sex. **(F)** TBX1 immunohistochemical staining of a representative scWAT image of female and a male participant. The magnification for all the pictures was 100 μm. ^∗^*p* < 0.05, ^∗∗^*p* < 0.01, and ^∗∗∗^*p* < 0.001.

Notably, *TBX1* mRNA scWAT expression increased significantly in the whole sample, in all groups (Nw, *p* < 0.05; Ow, *p* < 0.01; Ob *p* < 0.01) (Figure 3A) and in both sexes (female, *p* < 0.001; male, *p* < 0.01) (Figure 3B). When stratified by BMI, *CPT1B* mRNA expression significantly increased only in Ow subjects (*p* < 0.05). Interestingly, the acute irisin response showed a positive correlation with both Δ*CPT1B* and Δ*TBX1* expression in scWAT after the 12-week exercise intervention (*r* = 0.49 *p* < 0.05; *r* = 0.52 *p* < 0.01, respectively).

Multiple linear regression analyses showed that no browning gene expression changes were associated with insulin sensitivity improvement.

### Changes in Subcutaneous Adipose Tissue Immunochemistry

After the 12-week exercise intervention, adipocyte tissue UCP1 staining became positive (*p* < 0.01) (Figures 2C,D), and TBX1 (Figure 3C) and P2RX5 staining increased significantly in the entire sample (*p* < 0.001 and *p* < 0.01, respectively).

On stratifying by BMI groups and sex, TBX1 staining increased significantly in all BMI groups and in both sexes (Figures 3C–F), while UCP1 staining increased significantly only in the Ob group (*p* < 0.05) and in women (*p* < 0.05). Moreover, P2RX5 adipose tissue staining increased significantly in the Ow group (*p* < 0.05). CD38, CD137, SIGLEC8, ASC-1 and PAT2 immunostainings remained negative after exercise.

## Discussion

Brown adipose tissue has an extraordinary metabolic capacity to use glucose and free fatty acids for heat production and holds great promise in treating obesity, diabetes, and other metabolic disorders. Studies in humans have demonstrated that cold acclimatization increases BAT activity, leading to increased energy expenditure and improved glucose metabolism (Yoneshiro et al., 2013; Hanssen et al., 2015). However, humans have small amounts of BAT, and individuals with obesity and the elderly show even lower amounts that respond insufficiently to activation stimuli (Cypess et al., 2009; Carey et al., 2014; Chondronikola et al., 2014). Alternatively, WAT browning seems to be a more promising strategy for obesity treatment, where both pharmacological and physical activity approaches have been explored. In the present study, a 12-week exercise program induced scWAT gene expression and immunohistochemistry changes compatible with a browning process.

*UCP1* expression is known to be reduced in obesity (Cypess et al., 2009; Carey et al., 2014). Accordingly, we observed the lowest baseline *UCP1* expression levels in individuals with obesity, while the exercise intervention increased *UCP1* expression to levels comparable to those of Nw group. Although the mechanisms by which obesity inhibits *UCP1* expression remain unclear, a role of inflammation has recently been proposed, since integrin alpha 4 (ITGA4)- and VCAM-1 mediated macrophage-adipocyte interactions were found to inhibit *UCP1* expression by ERK1/2 and p38 pathway blockade. Moreover, *ITGA4* knockout mice and pharmacological *ITGA4* inhibition increased *UCP1* expression and subcutaneous adipose tissue browning in obese rodents (Chung et al., 2017). Our findings are in agreement with this, as scWAT *ITGA4* mRNA expression decreased and *UCP1* expression increased in the entire sample and in all BMI groups, particularly in the obesity group. This suggests that the known anti-inflammatory effect of exercise may revert *UCP1* inhibition in obese individuals (Kawanishi et al., 2010; Goh et al., 2016).

In accordance with previous studies in humans demonstrating that exercise promotes the expression of genes involved in oxidative phosphorylation (Rönn et al., 2014; Pino et al., 2016), the gene encoding fatty acid oxidation rate-limiting enzyme, CPT1B, was significantly up-regulated after the exercise intervention, mainly in overweight subjects. Twelve weeks of supervised physical activity also increased *TBX1* expression and immunostaining in all subjects, all groups and in both sexes. Interestingly, TBX1 is one of the few exclusive beige adipose tissue markers (Wu et al., 2012). While the role of *TBX1* in the adipocyte remains unclear, experimental evidence suggests it has a role in adipocyte dedifferentiation (Poloni et al., 2012). Remarkably, TBX1 immunostaining increased in adipocytes, predominantly in the stromal vascular fraction, which is consistent with a differentiation role in precursor cells. Together changes in these three markers (*UCP1, CPT1B* and *TBX1*), in addition to positive PR2X5 staining, a beige cell surface marker (Ussar et al., 2014), suggest a browning process after exercise.

In contrast with our observations, previous studies in humans have reported little or no effect of exercise on selected browning genes in scWAT (Norheim et al., 2014; Nakhuda et al., 2016; Pino et al., 2016; Tsiloulis et al., 2018). Several factors could explain these discrepancies. Firstly, most human studies have been performed in males. This could be relevant because our observations and those of a previous study (Scalzo et al., 2014) suggest that women have a greater browning response in white adipose tissue than men. Moreover, reports on exercise interventions in humans vary widely in type (endurance vs. resistance), intensity (60–80% HRmax), frequency (times per week) and duration of exercise routines (3 to 16 weeks). It has been previously stated that short duration of the interventions could explain the lack of exercise-browning effect (Pino et al., 2016). In contrast, a 6-month intervention of mild endurance exercise induced oxidative but not thermogenic markers in scWAT biopsies (Rönn et al., 2014), a pattern also reported in well-trained individuals (Pino et al., 2016). Human studies have also differed in the subcutaneous adipose tissue biopsy site (thighs, abdomen), known to show different gene expression patterns, adrenergic receptor quantities and lipolytic responses (Karastergiou et al., 2013; Fried et al., 2015; Passaro et al., 2017). Whether these differences might affect the browning capacity of different adipose tissue depots remains to be determined. Finally, there is wide inter-individual variability in response to exercise. Thus, mild effects such as exercise-induced *UCP1* up-regulation (∼1.65 fold in the present study, ∼1.82 fold as reported by Norheim et al., 2014) may not be evident in small sample studies. To date, large and highly significant scWAT browning effects (∼15-fold increase in *UCP1* expression) have been only observed in response to severe burn injury (Patsouris et al., 2015; Sidossis et al., 2015). Clearly severe burn injuries induce substantial and continuous catecholamine and cytokine release, in no way comparable to that induced by exercise routines.

In the present study, scWAT browning was not associated with insulin sensitivity improvement in the whole sample or in any BMI group. It is noteworthy that *UCP1* increased significantly after exercise only in individuals with obesity, who failed to show any metabolic improvement. While several studies in mice have shown that WAT browning improves metabolic parameters (Boström et al., 2012; Stanford et al., 2015b) and in humans, cold-activation of BAT ameliorates glucose metabolism and decreases body fat (van der Lans et al., 2013; Yoneshiro et al., 2013), evidence is insufficient to suggest that WAT browning has the same effects in humans. Still, burn injury-induced browning increases whole body metabolic rate, suggesting a positive effect of browning in humans (Patsouris et al., 2015; Sidossis et al., 2015). However, severe burning has a greater browning effect than exercise, which is probably the reason why we do not observed any association with metabolic improvement.

Whereas insulin sensitivity improvement was not associated with gene expression changes in scWAT, changes in adiponectin, irisin and apelin serum levels were the major insulin sensitivity determinants in a multiple linear regression analysis, predicting 60% of exercise-induced variation. Although the role of apelin and adiponectin in insulin sensitivity is well known, reports on the effect of exercise on circulating levels of adiponectin and apelin levels have been inconsistent (Jürimäe et al., 2006; Christiansen et al., 2010; Krist et al., 2013; Besse-Patin et al., 2014; Kelly et al., 2014; Bertrand et al., 2015). In contrast with some studies in humans reporting no change or increased adiponectin or apelin levels after an exercise intervention (Jürimäe et al., 2006; Christiansen et al., 2010; Kelly et al., 2014; Bertrand et al., 2015), we observed decreased adiponectin and apelin levels after exercise training in accordance with previous reports (Krist et al., 2013; Gastebois et al., 2016). Additionally, irisin, as part of the model, predicted insulin sensitivity improvement, in consistency with previous observations in mice (Boström et al., 2012). Irisin levels did not change significantly after the exercise program in accordance with previous studies (Hecksteden et al., 2013; Hofmann et al., 2014; Huh et al., 2014; Kurdiova et al., 2014), although there was a slight but significant increase in irisin levels only in the Ow group, the group showing the most significant improvement of metabolic parameters. Future studies in humans are required to fully elucidate the effect of exercise training on adipokines and myokines.

In our study, the age and BMI gaps were narrow and the intervention was well controlled. Nevertheless, the study had some limitations: firstly, because the sample size was reduced, stratification by BMI groups could have been underpowered to find significant differences. Moreover, diet was not controlled or assessed, and dietary factors could have influenced biochemical or other parameters. Additionally, there are still controversies regarding the measurement of irisin with commercial kits (Montes-Nieto et al., 2016). However, according to Perakakis et al. (2017) the ELISA kit used in our study is the best currently available (Perakakis et al., 2017). Development of more reliable assays and standardization of circulating irisin values are needed.

In conclusion, our findings demonstrate that 12-weeks of exercise training produced brown/beige gene expression changes in abdominal scWAT of non-diabetic individuals with different BMI. These changes did not contribute to the metabolic improvement, which should not be interpreted as a lack of effect of browning on metabolic parameters. These findings suggest that a bigger effect is needed and should not preclude the development of more effective strategies of browning. Furthermore, exercise-induced changes in adiponectin, apelin and irisin predicted insulin sensitivity improvement, supporting the important role of adipokines and myokines in metabolism homeostasis.

## Data Availability Statement

The raw supporting the conclusions of this manuscript will be made available by the authors, without undue reservation, to any qualified researcher.

## Author Contributions

BA-P, MR-F, EG-G, VS-M, and MS-S designed the project. MR-F and BO-D were responsible for individual recruitment and clinical assessment. MR-F, BO-D, and VS-M responsible for exercise supervision and individual follow-up. FM-P, SO-O, and MS-S performed the biopsies. BO-D, VB-E, and GB-G performed immunohistochemistry analyses. BO-D, BP-G, and RO-M carried out serum determinations and RT-PCR gene expression analyses. BO-D and BA-P performed data analysis. BO-D, MTV-M, and BA-P wrote the manuscript. All authors provided input to the manuscript, and all read and approved the final version.

## Conflict of Interest Statement

The authors declare that the research was conducted in the absence of any commercial or financial relationships that could be construed as a potential conflict of interest.
